# Acute appendicitis during the recovery phase of dengue hemorrhagic fever: two case reports 

**DOI:** 10.1186/s13256-022-03443-2

**Published:** 2022-06-05

**Authors:** V. Thadchanamoorthy, A. Ganeshrajah, Kavinda Dayasiri, N. P. Jayasekara

**Affiliations:** 1 Honorary Consultant Paediatrician, and Senior Lecturer, Faculty of Health Care Sciences, Eastern University, Batticaloa, Sri Lanka; 2 Actg. Consultant Paediatric Surgeon, Teaching Hospital, Batticaloa, Sri Lanka; 3 Consultant Paediatrician and Senior Lecturer, Faculty of Medicine, University of Kelaniya, Colombo, Sri Lanka; 4 Registrar in Paediatrics, Teaching Hospital, Batticaloa, Sri Lanka

**Keywords:** Dengue hemorrhagic fever, Acute appendicitis, Dengue shock syndrome, Tropical disease

## Abstract

**Background:**

Dengue fever is one of the most common tropical diseases, with high prevalence in many tropical countries including Sri Lanka. Dengue infection can present from subclinical infection to dengue shock syndrome. Further, the disease also shows a variety of atypical presentations and has been reported to mimic a number of causes of acute abdomen.

**Case presentation:**

The authors report two children (a 6-year-old Tamil girl and an 8-year-old Muslim girl) who were diagnosed to have acute appendicitis during the early recovery phase of dengue hemorrhagic fever (DHF) and late recovery period of dengue hemorrhagic fever with platelet count of 92 × 10^3^/cumm and 102 × 10^3^/cumm, respectively. Both children were investigated with abdomen ultrasound as they developed severe abdominal pain and tenderness on palpation during the recovery phase, which was felt to be very unusual. Acute appendicitis was diagnosed in one child, while the other child had a ruptured appendicular abscess. Both children were treated with laparoscopic appendectomy and a 7-day course of intravenous antibiotics. Both children were reviewed in 1 month following treatment and had complete recovery.

**Conclusion:**

Although precise pathophysiology and associations of the surgical abdomen with dengue fever remain to be elucidated, there are known factors in dengue fever that can potentially lead to secondary bacterial infections and surgical abdomen. Awareness and increased suspicion by the clinician are paramount to detect such complications early, especially in children who demonstrate unusual clinical features during various stages of dengue infection.

## Background

Dengue is a mosquito-borne viral infection and recognized as a common neglected tropical disease by the World Health Organization (WHO) [1]. The condition has had a great impact on the economy of Sri Lanka in providing both primary and secondary healthcare over the past two decades [2]. It has a myriad of clinical manifestations which range from self-resolving subclinical infection to fatal dengue shock syndrome [3]. Atypical presentations and multiorgan involvement were recognized under the umbrella of dengue expanded syndrome in 2012 by the WHO [4]. Various manifestations are reported in dengue expanded syndrome, including involvement of heart, brain, kidney, liver, spleen, pancreas, and gastrointestinal tract [5]. Reported uncommon complications are myocarditis [6], encephalitis [7], liver failure with encephalopathy [8], and acute kidney damage [9].

During dengue fever, abdominal pain is a common manifestation due to hepatic involvement, gastritis, myalgia, and hypovolemia [5]. Further, acute abdomen in the form of either medical or surgical complications is also a known presentation of dengue infection mimicking peritonitis, acute acalculous cholecystitis, acute pancreatitis, intussusception, and acute appendicitis [10–13]. The coexistence of dengue fever with the acute abdomen is undoubtedly a challenge to clinicians for timely diagnosis and treatment. Recognition of acute surgical conditions is mandatory to optimize appropriate treatment and reduce morbidity and mortality. The authors report two pediatric patients who manifested acute appendicitis during the recovery phase of dengue fever within a tertiary care setting during the heavy rainy season with an outbreak of dengue in eastern Sri Lanka.

## Presentation of case 1

A 6-year-old Tamil girl who had dengue aemorrhagic fever during the previous year was readmitted with fever, headache, vomiting, and abdominal pain for 5 days’ duration. This was the heavy rainy season with widespread dengue outbreaks in this region. She tested positive for dengue antigen (NS1) and had a low white blood cell count (2.8 × 10^3^/cumm), low platelet (70 × 10^3^/cumm), and hematocrit of 38, serum glutamic-oxaloacetic transaminase (SGOT) of 145 IU/dl, serum glutamic pyruvic transaminase (SGPT) of 98 IU/dl, and normal C-reactive protein. Her sister also had DHF and had been discharged recently.

Physical examination revealed that she was febrile (39 °C), appeared ill and irritable, and weighed 22 kg. All peripheral pulses were felt with low volume. Blood pressure was 90/70 mmHg with pulse pressure of 20 mmHg. Capillary refilling time was around 2 s with cold clammy extremities. Inward ultrasound revealed fluids in the Morrison pouch and right pleural space compatible with 8 h leakage time. The respiratory system had been unremarkable except for reduced air entry in the right lung base. There was generalized abdominal tenderness with 3-cm hepatomegaly. The rest of the system examination was normal.

**Fig. 1:**
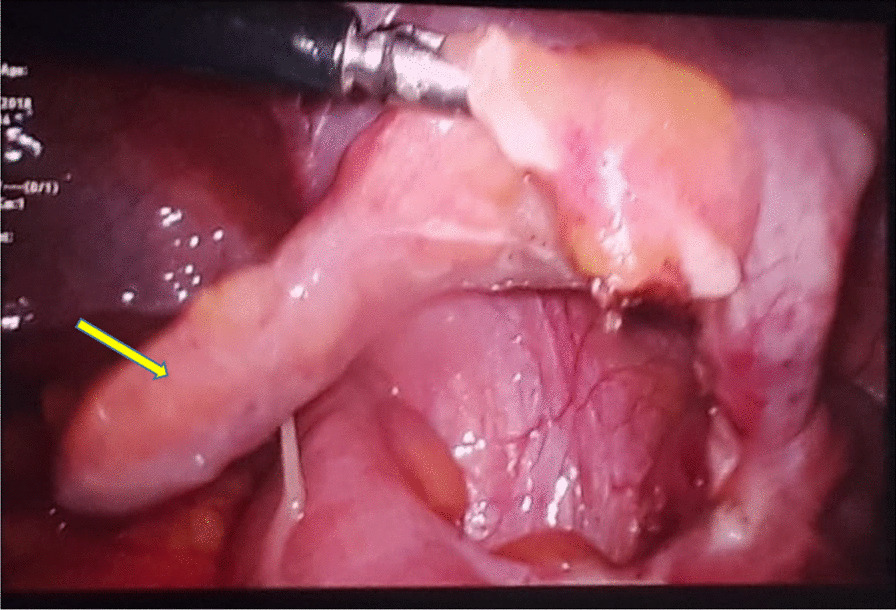
Laparoscopic view of acute appendicitis. Arrow indicates inflamed appendix

She was resuscitated with intravenous normal saline two boluses followed by 5 ml/kg/hour infusion. She improved with meticulous monitoring and fluid management. Her platelet count had dropped to 30 × 10^3^/cumm and gradually picked up on the seventh day of illness to 90 × 10^3^/cumm, with a WBC count of 4.5 × 10^3^/cumm, indicating recovery phase. The diagnosis of dengue fever was confirmed by the presence of dengue IgM and IgG antibodies, and her urine and blood cultures grew no organisms. On the night of the same day, she developed loose stools which were profuse, watery, and contained mucus. She also had vomiting and generalized abdominal pain. She was initially treated as having infective diarrhea pending stool cultures. However, stool culture was sterile after 48 hours of incubation. Repeated WBC count was 11 × 10^3^/cumm, and C-reactive protein was elevated to 12 mg/dL. The platelet count was 92 × 10^3^/cumm. She continued to have severe acute abdominal pain with demonstrable guarding and rigidity, and subsequently, repeated ultrasound in the late night was compatible with acute appendicitis. She underwent laparoscopic appendectomy and was given intravenous ampicillin 50 mg/kg/dose 6 hourly and metronidazole 7.5 mg/kg/dose three times a day for 7 days. Fig. 1 shows the appearance of the grossly inflamed appendix during laparoscopy. Several clinical follow-ups were performed at 1, 2, and 4 weeks after discharge, and the patient was noted to have made a complete recovery. The histopathological report confirmed the diagnosis of acute appendicitis.

## Presentation of case 2

**Fig. 2:**
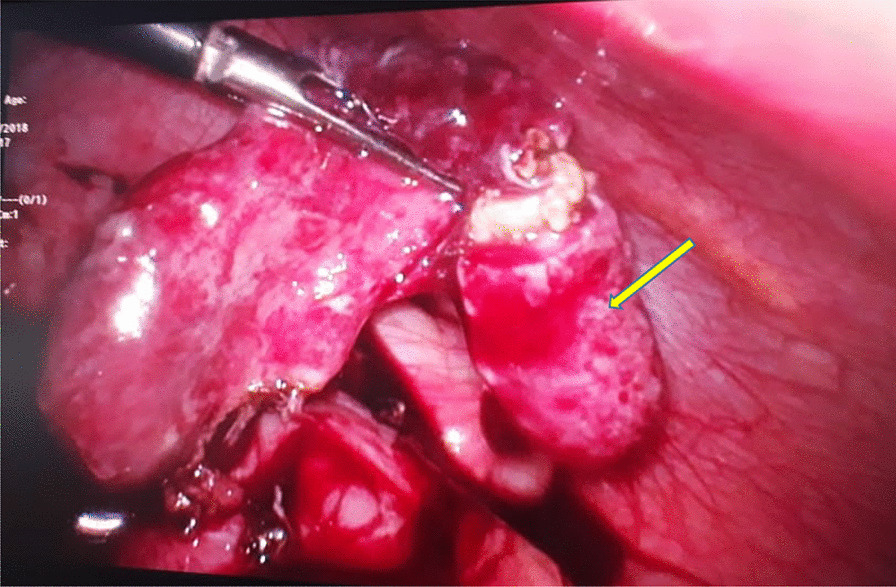
Laparoscopic view of the ruptured necrotic appendix with fecolith. Arrow indicates ruptured necrotic appendix with faecolith

An 8-year-old previously healthy Muslim girl, who had been treated for DHF in a local hospital, was transferred to the tertiary care unit for further management of abdominal pain of 2 days’ duration during a widespread outbreak of dengue infection while she had been in the late recovery period of DHF. She was admitted to the local hospital with fever which had been more than 38 °C, mild cough, and loose stools for 2 days. She was investigated with full blood count (lowest WBC count 3.2 × 10^3^/cumm, platelets 74 × 10^3^/cumm) and NS1 antigen (positive). Abdomen ultrasound initially showed pericolic collection of fluids, and she was managed as DHF with intravenous fluids and other supportive treatment. Urine, blood, and stool cultures grew no organisms. The diagnosis of dengue fever was confirmed by presence of dengue IgM and IgG antibodies. When she was planned for discharge with platelet count of 102 × 10^3^/cumm and WBC count of 8 × 10^3^/cumm on day 7 of illness, she developed vague generalized abdominal pain without tenderness. Ultrasound abdomen revealed that appendix was not visualized, but there were enlarged mesenteric multiple lymph nodes. She was managed symptomatically as mesenteric adenitis. She developed severe tenderness and abdominal pain in addition to severe vomiting and was transferred to the tertiary hospital for further management. Ruptured acute appendicitis was detected in a repeat ultrasound abdomen by the consultant radiologist. She was operated by laparoscopy; the image is shown in Fig. 2. Further treatment was intravenous cefotaxime at 50 mg/kg/dose, 6 hourly and metronidazole at 7.5 mg/dose, three times per day. Antibiotics were continued for 14 days as she had continued fever above 38.5 °C throughout the hospital stay. White blood cells (highest 24 × 10^3^/cumm) and C-reactive protein (highest 96 mg/dL) remained persistently elevated during the first week of intravenous antibiotics. Multiple clinical follow-up reviews were performed at 1, 2, and 4 weeks after discharge, and the patient was noted to have made a complete recovery with normal hematological and biochemical parameters. The histopathological report confirmed the diagnosis of acute appendicitis.

## Discussion

Dengue is one of the most common tropical diseases in Sri Lanka, presenting with abdominal pain and fever. Further, during dengue shock, hypovolemia also presents as severe abdominal pain [14]. Therefore, accurate and well-timed diagnosis of coexisting medical and surgical conditions such as acute appendicitis, acute pancreatitis, and cholecystitis is often difficult in dengue infection [11–13]. Children with predominant abdominal pain may be referred to surgeons initially, and dengue fever might be the underlying diagnosis subsequently, although it could potentially be detected with delay due to initial focus on the surgical abdomen [15]. Sometimes, an initial referral to the surgeon can lead to an unnecessary appendectomy [10]. Some authors suggest that acute abdomen may be a co-occurrence with dengue infection rather than a direct effect, although pathophysiological changes occurring in dengue may predispose to acute abdomen [15].

Lymphoid hyperplasia and mesenteric adenitis may also mimic acute appendicitis in dengue [16]. One of these reported children had enlarged multiple mesenteric lymph nodes in the first abdomen ultrasound, and subsequent ultrasound only showed ruptured acute appendicitis with abscess formation. The initial ultrasound findings misguided the clinical presentation as mesenteric adenitis and poorly visualized appendix instead of ruptured appendix. The pathophysiology of appendicitis in the context of dengue fever and its recovery is not clearly understood [17]. One potential etiology is lymphoid hyperplasia and mesenteric adenitis present in the febrile phase of dengue fever. The pathophysiological changes that occur during the onset of dengue hemorrhagic fever including systemic inflammatory response syndrome and plasma leakage are also likely to contribute to the development of acute abdomen including appendicitis [16]. Plasma leakage can result in an edematous appendix with luminal obstruction, promoting secondary bacterial infection and appendicitis [18]. Direct viral invasion can also lead to acute appendicitis. Other proposed mechanisms include endotoxemia and ischemic reperfusion injury [19].

A study in Pakistan showed that the incidence of acute abdomen in dengue fever had been 12% during the period of the dengue epidemic. Moreover, five of seven patients diagnosed with acute appendicitis underwent appendectomies, although histology did not favor their diagnosis. Therefore, accurate diagnosis is important to avoid unnecessary surgical procedures to reduce mortality [18]. A previously reported child in Sri Lanka revealed an appendicular mass occurring simultaneously with dengue infection [16]. Both of these children were diagnosed to have acute appendicitis during the recovery phase of DHF. The clinical presentation of case 1 was initially misinterpreted as acute bacillary dysentery due to high white blood cell count, high C-reactive protein, and normal abdomen ultrasound. However, careful consideration of alternative diagnoses and intense abdominal pain directed authors to repeat the ultrasound, which detected acute appendicitis. In the second case, although the first ultrasound was commented as acute mesenteric adenitis, increased abdominal pain and increased septic markers made the authors repeat the ultrasound by consultant radiologist.

Analyzing the clinical presentations of the two reported children in retrospect, it could be argued that the abdominal pain beyond the critical or viremia phase in dengue infection might be acute abdomen until proven otherwise. Therefore, an active survey of differential diagnoses is crucial to prevent morbidity and mortality of similar unexpected complications.

## Conclusion

Dengue fever is recognized to present with myriad atypical presentations, including the acute abdomen. Although precise pathophysiology and associations of the surgical abdomen with dengue fever remain to be elucidated, there are known factors in dengue fever that can potentially lead to secondary bacterial infections and surgical abdomen. Awareness and increased suspicion by the clinician are paramount to detect these complications early, especially in children, who demonstrate unusual clinical features during various stages of dengue infection.

## Data Availability

The data that support the findings of these case reports are available from Medical Records Department, Batticaloa Teaching Hospital, but restrictions apply to the availability of these data, which were used under license for the current report and so are not publicly available. Data are, however, available from the authors upon reasonable request and with permission of Medical Records Department, Batticaloa Teaching Hospital, Sri Lanka.

## Abbreviations

DHF: Dengue hemorrhagic fever
WHO: World Health Organization
WBC: Whole blood count
SGOT: Serum glutamic-oxaloacetic transaminase
SGPT: Serum glutamic pyruvic transaminase
